# Association of low-calorie sweetened product consumption and intakes of free sugar and ultra-processed foods in UK children: a national study from 2008 to 2019

**DOI:** 10.1007/s00394-025-03740-8

**Published:** 2025-06-26

**Authors:** Mathuramat Seesen, Kiara Chang, Jennie C. Parnham, Anthony A. Laverty, Christopher Millett, Fernanda Rauber, Renata B. Levy, Martin White, Edward W. Gregg, Eszter P. Vamos

**Affiliations:** 1 Public Health Policy Evaluation Unit, School of Public Health, Imperial College London, London, UK; 2 Public Health Research Centre and Comprehensive Health Research Centre, NOVA National School of Public Health, NOVA University Lisbon, Lisbon, Portugal; 3 Center for Epidemiological Research in Nutrition and Health, University of Sao Paolo, São Paulo, Brazil; 4 Preventive Medicine Department, Medical School, University of Sao Paolo, São Paulo, Brazil; 5 MRC Epidemiology Unit, School of Clinical Medicine, University of Cambridge, Cambridge, UK; 6 Department of Epidemiology and Biostatistics, School of Public Health, Imperial College London, London, UK; 7 School of Population Health, RCSI University of Medicine and Health Science, Dublin, Ireland; 8 University of Salamanca, Institute of Biomedical Research of Salamanca – IBSAL, Salamanca, Spain

**Keywords:** Low- and no-calorie sweetened products, Free sugar intake, Ultra-processed foods, UK children, Dietary trends

## Abstract

**Purpose:**

Little is known about the association between low- and no-calorie sweetened (LCS) product consumption and children’s dietary quality, despite the promotion of these products as sugar alternatives. This study examines the trends and associations between LCS product consumption and intakes of free sugar, ultra-processed food and beverages (UPFB), and other key dietary components among UK children.

**Methods:**

Repeated annual cross-sectional data from the National Diet and Nutrition Survey (2008/09-2018/19) for children aged 4–18 years (*N* = 5,922) were analysed. Children were categorized into No-LCS (0%g/day), Low-LCS (≤ 6.8%g/day), Mid-LCS (6.9–17.4%g/day), and High-LCS (≥ 17.4%g/day) groups based on a four-day food diary. The trends and associations were assessed using multivariable linear regression models, adjusted for sociodemographic factors.

**Results:**

In 2008/09, the High-LCS group had lower free sugar intake (-1.9%kcal_total_/day; 95% CI: -2.8, -1.0) compared with the No-LCS group. Over 11 years, free sugar intake declined in all groups, with similar declines observed across the LCS groups. By 2018/19, no difference in free sugar intake was observed between the High-LCS and No-LCS groups. Moreover, consumption of UPFB decreased (-0.8%g_total_/day per year; 95%CI: -1.1, -0.6) while water increased (2.7%g_drink_/day per year; 95%CI: 2.2, 3.1) in the No-LCS group over 11 years. Similar changes were observed in other LCS groups but were less pronounced in the High-LCS (vs. No-LCS) group.

**Conclusions:**

LCS product consumption was not consistently associated with lower free sugar intake among UK children. Differences in intakes of ultra-processed foods and water highlight the importance of considering dietary patterns beyond individual nutrients.

**Supplementary Information:**

The online version contains supplementary material available at 10.1007/s00394-025-03740-8.

## Introduction

Excessive free sugar intake is an independent risk factor for the development of cardiometabolic disorders including obesity, type 2 diabetes, and cardiovascular disease [1]. The average intake of free sugar among UK children is approximately 12% of the total dietary energy intake [2], two-fold higher than the maximum amount of 5% recommended by the UK Government and World Health Organization [3, 4]. Furthermore, one in three children aged 10 to 11 years have an unhealthy weight in England, with stark inequalities across levels of neighbourhood area deprivation [5].

Reducing population free sugar intake is a major public health priority to reduce childhood obesity in the UK [6], and sugar-sweetened beverages has been a key point of intervention as they contribute substantially to free sugar intake in UK children [7]. Industrial product reformulation to reduce sugar content in foods and beverages has been emphasised in flagship policies including the Soft Drinks Industry Levy [8] and the sugar reduction programme [9]. However, combined with strategies to encourage consumers to make ‘healthier food swaps’ such as the former Change4Life social marketing campaign [10] and the Government’s dietary recommendations [11], these efforts may result in the promotion of low- and no-calorie sweetened (LCS) products, such as those containing aspartame, acesulfame potassium, saccharin, sucralose, and steviol glycosides, as lower sugar alternatives.

The use of LCS have been heavily debated. Randomised controlled trials have suggested a short-term reduction in adiposity when substituting sugar-sweetened beverages by LCS alternatives in adults with overweight or obesity [12, 13]. However, longitudinal observational studies have linked LCS consumption to an increased risk of obesity [14], adverse cardiometabolic outcomes [14, 15], cancer [16], and mortality [17]. While mechanisms linking LCS consumption to an increased risk of cardiometabolic disorders are not fully understood, it has been suggested that early-life exposure to LCS may disrupt the physiological processes of sweet-taste response, appetite, and eating patterns during growth and development [18, 19]. Furthermore, it has been suggested that LCS might be associated with altered intestinal microbiota and chronic low-grade inflammation linked to obesity and impaired glucose metabolism [20].

Despite the uncertainties around the long-term health effects of LCS consumption, sales of LCS beverages in the UK have increased between 2015 and 2018 [21]. However, a few studies have examined the level of LCS product consumption in children and its association with nutritional and diet quality. Moreover, previous studies focused solely on LCS beverages, and their findings were mixed. One cross-sectional study of UK children found no difference in total energy and total sugar intake between LCS beverage consumers and non-consumers of both sugar-sweetened and LCS beverages [22]. The results of previous studies that assessed LCS beverage consumption in US children and their associations with free sugar intake and other indicators of dietary quality were mixed [23–25].

UK children have the highest levels of daily energy intake from ultra-processed foods in Europe [20], and ultra-processed dietary patterns have been linked to adverse health outcomes, including increased risk of adiposity and nutrient profiles associated with obesity in children [26], cardiometabolic diseases, and all-cause mortality [27]. Understanding how LCS product consumption relates to overall dietary patterns, including ultra-processed food and minimally processed food consumption alongside the intakes of key nutrients, is crucial for informing policies aimed at improving dietary quality and healthy weight in children. Therefore, this study aims to examine the trends and associations between the consumption of LCS products and intakes of free sugar, total energy, water, and ultra-processed foods in UK children aged 4–18 years, using nationally representative dietary survey data collected between 2008 and 2019. We hypothesized that higher levels of LCS product consumption form part of less favourable dietary patterns including higher levels of ultra-processed food and lower levels of minimally processed food consumption. We also hypothesized that LCS consumption would not be associated with free sugar intake.

## Methods

### Data source and study population

The UK National Diet and Nutrition Survey (NDNS) from year 1 (2008–2009) to year 11 (2018–2019) were used in this study, and the methodological details on data collection can be found elsewhere [28]. In brief, the NDNS is an annual cross-sectional survey designed to collect food and nutritional intake from representative participants aged 1.5 years and above, including approximately 500 children and 500 adults across the UK. The survey applied a stratified random sampling which was selected individuals living in private households based on postcodes. Computer assisted personal interviews were conducted to collect socio-demographic information of the participants. Participants (or guardians of those aged 12 years or under) completed a four-day (non-consecutive) food diary recording everything they ate and drank, together with the portion size, cooking and seasoning methods, and brand names. Nutritional composition of foods and beverages were obtained through data linkage to the Department of Health’s NDNS Nutrient Databank [29].

In this study, we considered data from all children aged between 4 and 18 years who participated within any of the year 1–11 of NDNS waves for inclusion. Four children with missing ethnicity data and seven children with missing Index of Multiple Deprivation (IMD) data were excluded from the analysis. Children with missing BMI were assigned to a ‘missing’ category (*N* = 290, 4.9%) for the purpose of preserving sample size. This resulted in a total of 5,922 children included in the analytical sample.

### Study exposure

Participants’ consumption of LCS products was calculated from the food diaries. We screened the food names of 5,196 unique food and beverage items ever consumed by NDNS participants, and a total of 140 items were categorised as LCS products, consisting of 53 foods and 87 beverages. Further details are provided in Appendix Fig. 1.

For each participant, we computed the percentage of the amount of LCS product (in grams) consumed relative to the total amount of food and beverages (in grams) consumed in a day and averaged across multiple days (5,813 participants completed 4-day and 109 participants completed 3-day food diary).

We categorized participants into four groups according to the levels of LCS product consumption. We defined No-LCS group as participants with zero consumption of LCS products (0% g/day), and Low-LCS, Mid-LCS, and High-LCS groups representing the tertiles of those with some LCS consumption (> 0% g/day). The tertiles were derived based on the distribution of LCS product consumption among those participated in the first year of survey 2008–2009. Therefore, the study population with LCS product consumption ≤ 6.8% g/d, between 6.9 and 17.4% g/d, and ≥ 17.4% g/d, were subsequently categorised into Low-LCS, Mid-LCS, and High-LCS groups, respectively (Appendix Fig. 2).

### Outcomes

We considered participants’ free sugar and dietary energy intake as primary outcome measures, and secondary outcome measures included dietary intakes of total sugar, water, and the consumption of minimally processed and ultra-processed foods and beverages in the diet. We have previously applied the Nova food classification system to NDNS data categorising each food and beverage into one of the four mutually exclusive food groups based on the extent and purpose of the food processing they undergo [30]. The minimally processed and ultra-processed food groups represent the lowest and highest degree of food processing defined by the Nova classification accordingly. Further details on the definition of the Nova classification are provided in Appendix Table 1.

Total daily energy intake was measured in kilocalorie (kcal) of the total diet (kcal/d), averaged across multiple days. Free sugar and total sugar intake were measured by the proportion of total energy from all foods and beverages consumed (%kcal_total_/d) and from drinks specifically (%kcal_drinks_/d); as well as by the total intake from all foods and beverages consumed (grams per day; g/d) and from drinks specifically (g/d). Water intake was measured by the proportion of total grams intake from drinks (%g_drinks_/d) and by the total amount consumed in grams (g/d). Consumption of ultra-processed foods and beverages, ultra-processed foods (excluding beverages), and minimally processed foods and beverages were measured by the proportion of total grams intake from all foods and beverages consumed (%g_total_/d) and by the total amount consumed in grams (g/d).

### Covariates

Study covariates include age, sex (boys, girls), ethnicity (white, non-white), equivalized household income tertiles (highest, middle, lowest), IMD quintile, and body mass index (BMI; normal weight, overweight, obese). IMD refers to a measure of relative deprivation assigned to small areas of the UK, based on seven domains, including income, employment, education, health, crime, housing and services, and living environment [31]. Height and weight were objectively measured in the NDNS, and the classification of BMI categories was provided based on the age-sex specific BMI centiles according to the British 1990 growth reference [32].

### Statistical analysis

Characteristics of the study participants were compared among LCS consumption groups within each yearly cohort using Kruskal-Wallis test for continuous and Chi-square test for categorical variables. Furthermore, characteristics of the study cohort from the first (2008–2009) and last (2018–2019) survey years were compared using Wilcoxon rank sum test for continuous and chi-square test for categorical variables.

Dietary intakes of total energy, energy from free sugar, and energy from total sugar that contribute to each Nova subgroup were calculated and displayed graphically by LCS consumption groups. Multivariable linear regression was used to examine the trend and association between LCS consumption groups and each study outcome. The regression models included a continuous year variable to examine trends and an interaction term between the year and LCS consumption group variables to assess differences in study outcomes between LCS consumption groups. The models were fully adjusted for all study covariates and the NDNS survey weights were applied [29]. Bonferroni correction was applied to account for multiple comparisons. All analyses were performed using R version 4.2.2. Statistical significance was defined as a 2-tailed P-value below 0.05.

## Results

Over the study period between 2008 and 2009 and 2018–2019, a total of 5,922 children were included in the analyses (ranging between 424 and 718 children annually). Participants’ characteristics by the levels of their LCS consumption for the first and last survey years are presented in the Table 1. In 2008–2009, the median age of children was 10 years (IQR, 7.0 to 14.0), 49.2% were boys, 89.0% were of white ethnicity, 64.9% had a BMI in the normal range, and 36.2% belonged to the lowest household income tertile (Table 1). Moreover, children in the High-LCS group were younger and more likely to be of white ethnic group than those in other groups. Across survey years, the characteristics of children in all LCS consumption groups remained largely similar, except for a lower proportion of participants with white ethnic background (89.0% in 2008–2009 vs. 82.3% in 2018–2019; *P* < 0.01), and a larger proportion with a missing BMI (4.8% vs. 8.3%; *P* = 0.03).

The proportional distribution of children in each group and their mean intake of LCS products have not substantially changed throughout the 11-year study period (Appendix Fig. 2). In 2008–2009, 70.4% of participants consumed LCS products, with a mean intake of 256.5 g/d among consumers. The prevalence and mean intake, both overall and within each LCS group, did not significantly change by 2018–2019 (Appendix Table 2).

**Fig. 1:**
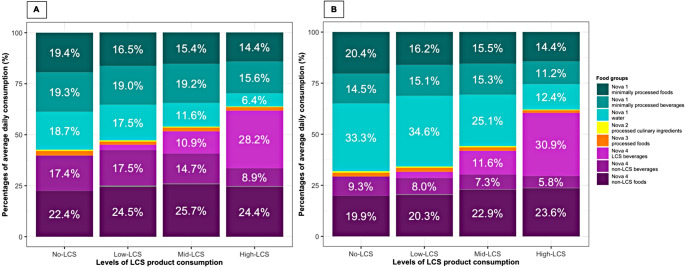
Grams of dietary intake by Nova subgroup in 2008–2009 (**A**) and 2018–2019 (**B**). **A** (*N* = 646). Percentages of dietary intake by Nova subgroups. No-LCS: Nova 2 processed culinary ingredients, 0.4%; Nova 3 processed foods and beverages, 2.3%. Low-LCS: Nova 2 processed culinary ingredients, 0.5%; Nova 3 processed foods and beverages, 1.6%; Nova 4 LCS beverages, 2.6; Nova 4 LCS foods, 0.5%. Mid-LCS: Nova 2 processed culinary ingredients, 0.4%; Nova 3 processed foods and beverages, 1.8%; Nova 4 LCS foods, 0.4%. High-LCS: Nova 2 processed culinary ingredients, 0.3%; Nova 3 processed foods and beverages, 1.7%; Nova 4 LCS foods, 0.3%. **B** (*N* = 424). Percentages of dietary intake by Nova subgroups. No-LCS: Nova 2 processed culinary ingredients, 0.6%; Nova 3 processed foods and beverages, 2.0%. Low-LCS: Nova 2 processed culinary ingredients, 0.5%; Nova 3 processed foods and beverages, 2.1%; Nova 4 LCS beverages, 3.0; Nova 4 LCS food, 0.2%. Mid-LCS: Nova 2 processed culinary ingredients, 0.5%; Nova 3 processed foods, 1.7%; Nova 4 LCS foods, 0.1%. High-LCS: Nova 2 processed culinary ingredients, 0.3%; Nova 3 processed foods, 1.3%; Nova 4 LCS foods, 0.1%

Figure 1 illustrates the mean dietary contribution of each Nova subgroup relative to the total food and beverage intake (%g_total_/day) stratified by LCS consumption groups in 2008–2009 and 2018–2019. In 2008–2009, a gradient of lower water intake was observed with increasing levels of LCS consumption from 18.7%g_total_/day to 6.4%g_total_/day. Compared with the No-LCS group, children in the High-LCS group had a higher intake of non-LCS Nova 4 foods but a lower intake of both Nova 1 beverages and non-LCS Nova 4 beverages. In 2018–2019, the overall patterns remained largely similar to that observed in 2008–2009. However, the proportional intake of water increased in all groups, and the consumption of minimally processed beverages reduced during the study period. There was also a general reduction.

in the proportional intake of non-LCS ultra-processed drinks and a decrease in the proportional intake of non-LCS ultra-processed foods.

**Table 1:** Characteristics of the study population in 2008–2009 (*N* = 646) and 2018–2019 (*N* = 424)

Characteristics	NDNS year 2008–2009						NDNS year 2018–2019						2008–2009 vs.
	(*n* = 646)						(*n* = 424)						2018–2019
	Total (*n* = 646)	No-LCS (*n* = 191)	Low-LCS (*n* = 152)	Mid-LCS (*n* = 151)	High-LCS (*n* = 152)	*P*-value	Total (*n* = 424)	No-LCS (*n* = 133)	Low-LCS (*n* = 86)	Mid-LCS (*n* = 85)	High-LCS (*n* = 120)	*P*-value	*P*-value
Age, median (IQR)	10 (7.0, 14.0)	11 (7.0, 14.5)	10 (7.0, 14.0)	10 (8.0, 14.0)	9 (6.0,13.0)	0.049*	10 (7.0, 13.0)	11 (7.0, 15.0)	11 (9.0, 14.0)	11 (7.0, 13.0)	9 (6.0, 12.0)	< 0.01	0.9
Boys, n (%)	318	100	69	74	75	0.7	212	54	42	43	73		
	(49.2)	(52.4)	(45.4)	(49.0)	(49.3)		(50.0)	(40.6)	(48.8)	(50.6)	(60.8)	0.02	0.9
White ethnic group, n (%)	575.0 (89.0)	155	131	141	148	< 0.001^+^	349	93	67	77	112		
		(81.2)	(86.2)	(93.4)	(97.4)		(82.3)	(69.9)	(77.9)	(90.6)	(93.3)	< 0.001^+^	< 0.01**
Household income						0.4						0.8	0.2
tertile, n (%)													
- Highest	169 (26.2)	55 (28.8)	33 (21.71)	44 (29.1)	37 (24.3)		111 (26.2)	39 (29.3)	24 (27.9)	20 (23.5)	28 (23.3)		
- Middle	166 (25.7)	46 (24.1)	36 (23.7)	42 (27.8)	42 (27.6)		130 (30.7)	39 (29.3)	30 (34.9)	27 (31.8)	34 (28.3)		
- Lowest	234 (36.2)	62 (32.5)	64 (42.1)	54 (35.8)	54 (35.5)		131 (30.9)	39 (29.3)	20 (23.3)	29 (34.1)	43 (35.8)		
- Missing value	77 (11.9)	28 (14.7)	19 (12.5)	11 (7.3)	19 (12.5)		52 (12.3)	16 (12.0)	12 (14.0)	9 (10.6)	15 (12.5)		
IMD, n (%)						0.9						0.52	0.8
- Quintile 1	144 (22.3)	43 (22.5)	37 (24.3)	32 (21.2)	32 (21.1)		88 (20.8)	27 (20.3)	15 (17.4)	23 (27.1)	23 (19.2)		
- Quintile 2	134 (20.7)	40 (20.9)	32 (21.1)	33 (21.9)	29 (19.1)		89 (21.0)	23 (18.3)	20 (23.3)	18 (21.2)	28 (23.3)		
- Quintile 3	113 (17.5)	29 (15.2)	27 (17.8)	33 (21.9)	24 (15.8)		79 (18.6)	20 (15.0)	16 (18.6)	16 (18.8)	27 (22.5)		
- Quintile 4	111 (17.2)	33 (17.3)	25 (16.5)	26 (17.2)	27 (17.8)		81 (19.1)	27 (20.3)	16 (18.6)	16 (18.8)	22 (18.3)		
- Quintile 5 (least deprive)	144 (22.3)	46 (24.1)	31 (20.4)	27 (17.9)	40 (26.3)		87 (20.5)	36 (27.1)	19 (22.1)	12 (14.1)	20 (16.7)		
BMI, n (%)						0.69						0.27	0.04*
- Normal	419 (64.9)	132 (69.1)	93 (61.2)	100 (66.2)	94 (61.8)		270 (63.7)	91 (68.4)	58 (67.4)	44 (51.8)	77 (64.2)		
- Overweight	86 (13.3)	22 (11.5)	27 (17.8)	17 (11.3)	20 (13.2)		45 (10.6)	16 (12.0)	8 (9.3)	9 (10.6)	12 (10.0)		
- Obese	110 (17.0)	30 (15.7)	23 (15.1)	27 (17.9)	30 (19.7)		74 (17.5)	17 (12.8)	15 (17.4)	20 (23.5)	22 (18.3)		
- Missing value	31 (4.8)	7 (3.7)	9 (5.9)	7 (4.6)	8 (5.3)		35 (8.3)	9 (6.8)	5 (5.8)	12 (14.1)	9 (7.5)		

* P-value < 0.05, ** P-value < 0.01, ^+^ P-value < 0.001

**Statistical analysis**: Kruskal-Wallis test was used to compare the distribution of age across different consumption levels of AS products. Wilcoxon rank sum test was used to compare age between year 1 and year 11. Chi-square test was used to compare the proportions of categorical variables across different consumption levels of LCS products and between year 1 and year 11

### Free sugar and total energy intake from 2008–2009 to 2018–2019

**Fig. 2:**
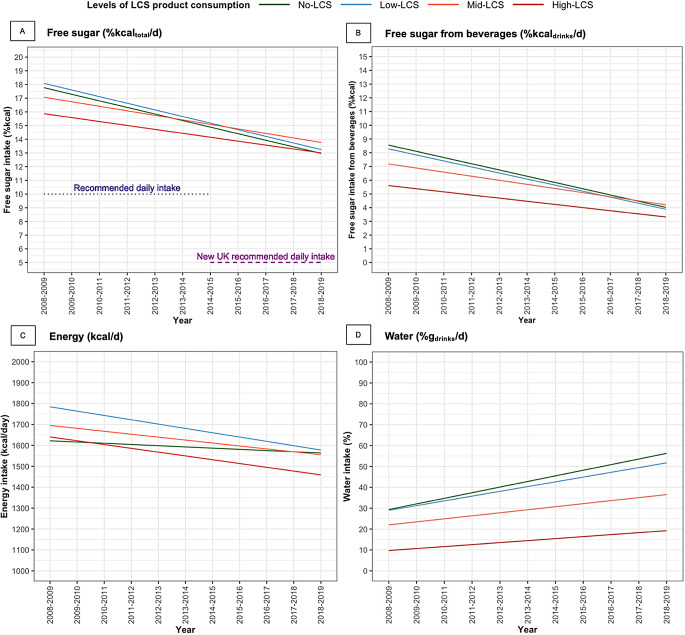

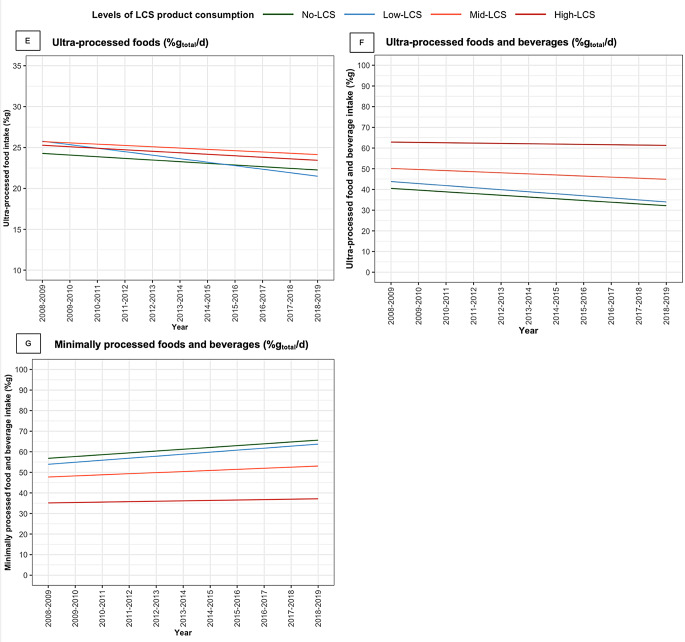
Trends in daily dietary component intake by levels of LCS product consumption among UK children

Results of multivariable linear regression showed that in 2008–2009, the proportion of total energy intake from free sugars was − 1.9%kcal_total_ (95% CI, -2.8, -1.0) lower among children in the High-LCS group compared with those in the No-LCS group and − 3.0%kcal_drinks_ (95% CI, -3.7, -2.2) lower for free sugars from beverages (Table 2; Fig. 2). Free sugar intake in the No-LCS group showed a downward trend by 0.5%kcal_total_ reduction per year (95% CI, -0.6 to -0.4) for intakes from the overall diet and 0.5%kcal_drinks_ reduction per year (95% CI -0.5 to -0.4) from beverages. The High-LCS group showed a smaller decline in free sugar intake from both overall diet and beverages compared with the No-LCS group. However, after Bonferroni correction, the difference in free sugar intake from overall diet no longer reached statistically significance. By year 2018–2019, there were no statistically significant differences in free sugars intake from neither the overall diet nor beverages between No-LCS and High-LCS groups (Appendix Table 4). When considering the free sugar intake measured in grams consumed, the trends and associations were consistent with the findings of the relative measures (Appendix Table 3, Appendix Fig. 3). Furthermore, findings for total sugar intake were similar to those from free sugar intake.

In year 2008–2009, the mean total energy intake (kcal/day) was significantly higher among the Low-LCS and Mid-LCS groups but similar for the High-LCS group as compared with the No-LCS group (Table 2; Fig. 2). The trend in total energy intake in the No-LCS group did not significantly change over time. However, the total energy intake for the Low-LCS and High-LCS groups showed a decline, with an incremental yearly reduction of 14.8 kcal/day (95% CI, -25.4 to -4.3) and 12.3 kcal/day (95% CI, -22.5, -2.0), respectively compared with the No-LCS group.

**Table 2:** Association between daily LCS product consumption and dietary components from 2008–2019 (*n* = 5,922)

Intake of nutrients	Free sugar intake (%kcal_total_/d)	Free sugar intake from beverages (%kcal_drinks_/d)	Total energy intake (kcal/d)	Water intake (%g_drinks_/d)	Ultra-processed food and beverage intake (%g_total_/d)	Ultra-processed food intake (%g_total_/d)	Minimally processed food and beverage intake (%g_total_/d)
	Coefficient (95% CI)	Coefficient (95% CI)	Coefficient (95% CI)	Coefficient (95% CI)	Coefficient (95% CI)	Coefficient (95% CI)	Coefficient (95% CI)
LCS product consumption at year 1^**+**^							
- No-LCS	Reference	Reference	Reference	Reference	Reference	Reference	Reference
- Low-LCS	0.3 (-0.7, 1.3)	-0.3 (-1.2, 0.6)	162.4 (98.3, 226.5)*^+^	-0.4 (-4.3, 3.5)	3.4 (0.8, 5.8)*	1.5 (0.2, 2.8)*	-2.9 (-5.3, -0.4)*
- Mid-LCS	-0.7 (-1.7, 0.3)	-1.4 (-2.2, -0.6)*^+^	73.8 (2.9, 144.7)*^+^	-7.3 (-11.0, -3.8)*^+^	9.6 (7.3, 12.0)*^+^	1.4 (0.1, 2.8)*	-9.1 (-11.4, -6.7)*^+^
- High-LCS	-1.9 (-2.8, -1.0)*^+^	-3.0 (-3.7, -2.2)*^+^	18.6 (-42.3, 79.4)	-19.6 (-22.7, -16.6)*^+^	22.4 (20.1, 24.6)*^+^	1.0 (-0.3, 2.2)	-21.7 (-23.9, -19.4)*^+^*
Year	-0.5 (-0.6, -0.4)*^+^	-0.5 (-0.5, -0.4)*^+^	-5.8 (-13.2, 1.5)	2.7 (2.2, 3.1)*^+^	-0.8 (-1.1, -0.6)*^+^	-0.2 (-0.3, -0.1)*	0.9 (0.6, 1.1)*^+^
Interaction							
- No-LCS x Year	Reference	Reference	Reference	Reference	Reference	Reference	Reference
- Low-LCS x Year	-0.003 (-0.2, 0.2)	0.01 (-0.1, 0.2)	-14.8 (-25.4, -4.3)*^+^	-0.4 (-1.1, 0.3)	-0.1 (-0.6, 0.3)	-0.2 (-0.5, 0.002)	0.1 (-0.3, 0.5)
- Mid-LCS x Year	0.5 (-0.01, 0.3)	0.2 (0.02, 0.3)*	-8.2 (-20.2, 3.7)	-1.2 (-1.9, -0.6)*^+^	0.3 (-0.1, 0.7)	0.04 (-0.2, 0.3)	-0.4 (-0.7, 0.03)
- High-LCS x Year	0.2 (0.1, 0.3)*	0.2 (0.1, 0.3)*^+^	-12.3 (-22.5, -2.0)*^+^	-1.7 (-2.3, -1.2)*^+^	0.7 (0.3, 1.1)*^+^	0.02 (-0.2, 0.2)	-0.7 (-1.1, -0.3)*^+^

* P-value < 0.05 before Bonferroni correction, *^+^ P-value < 0.05 after Bonferroni correction

^**+**^ The levels of LCS product consumption represent nutritional outcome in children relative to group1in 2008–2009

**Statistical analysis**: Multivariable linear regression adjusted for age, sex, ethnicity, household income, Indices of Multiple Deprivation, and body mass index

### Water intake from 2008–2009 to 2018–2019

The fully adjusted models showed that compared with the No-LCS group in 2008–2009, the mean proportion of water intake among all beverages consumed was significantly lower in Mid-LCS and High-LCS groups (Table 2; Fig. 2). Over the 11-year study period, children in the No-LCS group experienced a significant increase in daily water intake, by a yearly increment of 2.7%g_drinks_ (95% CI, 2.2 to 3.1). Trends in daily water intake increased similarly in children of higher LCS groups, but the annual increments were smaller in Mid-LCS and High-LCS groups by -1.2%g_drinks_ (95% CI, -1.9, -0.6) and − 1.7%g_drinks_ (95% CI, -2.3 to -1.2) compared with children in No-LCS group, respectively.

### Ultra-processed and minimally processed food intake from 2008–2009 to 2018–2019

Over 11 years, children in the No-LCS group showed an annual decrease in the proportion of ultra-processed foods and beverages consumed by -0.8%g_total_/day (95% CI, -1.1, -0.6) (Table 2; Fig. 2). By contrast, there was a smaller decrease in children in the High-LCS compared with the No-LCS group by 0.7%g_total_/day (95% CI, 0.3, 1.1). When considering only the consumption of ultra-processed foods (excluding beverages), Bonferroni-corrected results showed no significant difference in mean daily intake across LCS groups in 2008–2009, nor any statistically significant changes in consumption over 11 years within any of the LCS consumption groups.

The consumption of minimally processed foods and beverages in the No-LCS group increased significantly by 0.9%g_total_/day (95% CI 0.6 to 1.1) annually (Table 2; Fig. 2). This trend was not significantly different among children in the Low-LCS and Mid-LCS groups. However, the annual increase in the High-LCS group was smaller by -0.7%g_total_/day (95% CI -1.1 to -0.3) compared with the No-LCS group.

The trends and associations between the LCS consumption groups and the absolute intakes of total energy, ultra-processed foods and beverages, and minimally processed foods and beverages are presented in the Appendix Table 3. The findings were largely consistent with those presented in the main results.

## Discussion

This nationally representative study examined the associations of LCS product consumption and key dietary intakes of UK children between 2008 and 2019. About 70% of children consumed LCS products in 2008–2009, and this level of LCS product consumption remained largely unchanged during the study period. Our study presents four notable findings. First, children with the higher levels of LCS product consumption had lower intakes of water, minimally processed beverages, and non-LCS ultra-processed beverages compared with non-consumers of LCS products. Second, they also had lower intakes of free sugar and total sugar in 2008–2009 compared with non-consumers of LCS products, but this difference diminished by the end of the study period. Third, there was a greater decline in the total energy intake among children with the highest LCS product consumption compared with non-consumers of LCS products. Finally, all LCS consumption groups had similar declines in the consumption of ultra-processed foods and beverages over the study period, except for the High-LCS group whose decline was less pronounced.

### Comparison with previous studies

Temporal changes in free sugar intake have not been examined previously among children with different levels of LCS consumption in the UK. While according to the best of knowledge, no study has analysed the consumption of LCS foods in children, a few studies have assessed the association between the consumption of LCS beverages and dietary quality in children, with mixed findings. A previous UK study that pooled NDNS data between 2008 and 2012 did not observe differences in total energy and total sugar intake between LCS beverage consumers and non-consumers of both sugar-sweetened beverages and LCS beverages [22]. By contrast, a pooled cross-sectional study of US children found that those who consumed LCS beverages had higher intakes of total energy, total sugar, and added sugar compared with those who consumed water predominantly [23].

Reducing population free sugar intake in children has been at the forefront of public health policy in the UK [6], and the existing policies often encourage the substitution of sugar-sweetened products by LCS alternatives. Our study shows that two-thirds of children consumed LCS products in the UK, and this level of LCS product consumption remained consistently high throughout the 11-year study period. Children in the highest LCS consumption group had lower free sugar intake than non-consumers of LCS products in 2008–2009. However, the diminishing difference in free sugar intake between the High-LCS and No-LCS groups by 2018–2019 raises the question of whether the promotion of LCS as alternatives to sugar-sweetened products is an effective public health strategy to reduce free sugar intake in children and improve overall dietary patterns.

This study observed a decline in the consumption of ultra-processed foods and beverages over the study period. This finding aligns with a previous study that analysed NDNS data from adolescents from 2008 to 2019, reporting a decrease in ultra-processed food and beverage consumption over 11 years [33]. Our study further demonstrates that children with the highest LCS product consumption exhibited a slower decline in ultra-processed food and beverage consumption as well as a slower increase in minimally processed food and beverage consumption compared with non-consumers of LCS products throughout the study period. These trends suggest that LCS products may be key components of an ultra-processed dietary pattern and could partly explain the smallest improvement in water intake observed among children with the highest LCS product consumption relative to the other groups. These findings also highlight the importance for future research to examine the consumption of LCS products in children and for public health policies aimed at improving children’s diet to sufficiently consider the evidence and debate on the health risks and benefits of LCS products.

We have found that water consumption in UK children has increased over the 11-year study period. However, this change was less pronounced in children with the highest LCS consumption. Water is the main source of hydration recommended for children, and the potential substitution of sugar-sweetened beverages with LCS beverages may present a missed opportunity to increase water consumption in children. Importantly, substitution of sugar-sweetened beverages by water has been shown effective in reducing children’s total energy intake [34]. In addition, randomised controlled trials have reported that drinking water can improve cognitive performance in children [35, 36]. A study in adults has also demonstrated that replacing sugar-sweetened beverages with water sustains lower calorie intake over 12 months, while the same effect was not observed when replacing sugar-sweetened beverages with LCS beverages [37]. Future research that examines water intake in children and evaluates substitution patterns when reducing sugar-sweetened beverage consumption in children should also consider the intake of LCS beverages along with the overall dietary pattern including ultra-processed and minimally processed foods and beverages.

### Strengths and limitations

Our study has several strengths. This is the first study to analyse the trends in consumption of key nutrients and dietary components among children in the UK by levels of LCS product consumption. We utilised data from the NDNS, which is nationally representative with dietary intake data collected through a detailed 4-day food diary that captured all foods and beverages consumed. The findings of this study may be primarily generalisable to children living in high-income countries with comparable dietary environments and public health policies that may influence the consumption of LCS products. However, while food environment and policy and social-cultural contexts may differ across countries, the global rise in LCS product consumption and their increasing use as alternatives to sugary products highlights the importance of evaluating children’s LCS consumption in conjunction with overall dietary patterns and free sugar intake.

There are limitations to consider. First, the amount of LCS product consumption in the study population may have been underestimated because of no ingredient list is available in the NDNS data. The tertiles of LCS consumption were based on the proportion of LCS products consumed in the total diet, and we could not verify the accuracy of this approach since there were no information on the amount of low- and no-calorie sweeteners consumed in diet. Second, LCS beverages in powder or concentrated form were analysed in their recorded weight as the amount of liquid diluent could not be accurately estimated. Third, the study is limited by unobserved (e.g. adverse childhood events [38]) and residual confounding due to observational nature of the data. Fourth, this study was unable to provide data on individual changes in dietary consumption as the NDNS data are serial cross-sectional. Self-reported dietary intake data are subject to recall bias and misreporting. However, the NDNS has several methodological strengths to reduce bias. These include the use of 4-day food diaries combined with interviewer-administered methods, portion-size estimation tools, biological samples for cross-validation of dietary data, standardised protocols, and rigorous quality control measures.

## Conclusions

In this nationally representative study of UK children, we found that higher LCS product consumption was not consistently associated with lower free sugar intake in children. Furthermore, LCS product consumption may be associated with lower intakes of water and other minimally processed foods in children’s diet. These findings highlight the need for more comprehensive consideration of dietary patterns, beyond individual nutrients.

## Electronic supplementary material

Below is the link to the electronic supplementary material.

Supplementary Material 1

## Data Availability

The datasets supporting the conclusion of this article are available upon request via UK Data Service website (https://ukdataservice.ac.uk/).

## Abbreviations

UK: United Kingdom
LCS: Low and no-calorie sweetened
NDNS: National Diet and Nutrition Survey
IMD: Index of Multiple Deprivation
G: Grams
Kcal: Kilocalories
