# Supplementary material for: Association of low-calorie sweetened product consumption and intakes of free sugar and ultra-processed foods in UK children: a national study from 2008 to 2019
Source: Eur J Nutr. 2025 Jun 26;64(5):230. doi: 10.1007/s00394-025-03740-8 (PMC12202694; doi:10.1007/s00394-025-03740-8)
Supplement: Supplementary file 1 — Supplementary Material 1 [file 394_2025_3740_MOESM1_ESM.docx]

**Supplementary appendix**

**Appendix Table 1** Description of the subsidiary group within the Nova food classification

| Food group | Description |
| --- | --- |
| Nova 1 minimally processed foods and beverages | Foods and beverages that have either not been modified or have only undergone processes to preserve or make them edible, squeezing, boiling, pasteurization, chilling, and freezing, These processes do not involve the addition of salt, sugar, oil, or other substances. Examples include grains, fruits, vegetables, meat, fish, seafood, eggs, plain water, coffee, tea, fresh fruit juices, and milk. |
| Nova 2 processed culinary ingredients | Ingredients used for seasoning and cooking, extracted from Nova 1 foods or sourced from nature; for example, sugar, salt, vegetable oils, butter, and honey. |
| Nova 3 processed foods | Foods and beverages produced by adding salt, sugar, oil, or other Nova 2 ingredients to Nova 1 foods. The processes may include canning, bottling, and alcoholic fermentation. Examples include canned meat or fish, canned vegetables or fruits, cure meat, cheeses, beer, wine, and bread that make from ingredients that used in household culinary preparations. |
| Nova 4 ultra-processed foods and beverages | Foods and beverages that are formulations of ingredients derived from extensive industrial processing, such as ready-to-eat and ready-to-heat dishes, packaged snacks, mass-produced packaged bakery, margarine, breakfast cereal, flavored yogurt, confectioneries, carbonated drinks, flavored drinks, energy drinks, flavored milk or yogurt drinks, and products containing low- or no-calorie sweeteners. |

**Appendix Table 2 Average consumption of LCS products among UK children by year of NDNS data collection**

| Levels of LCS food and beverage consumption | Average daily consumption (grams), median (IQR) | | | | | | | | | | | P-value |
| --- | --- | --- | --- | --- | --- | --- | --- | --- | --- | --- | --- | --- |
|  | 2008-2009 | 2009-2010 | 2010-2011 | 2011-2012 | 2012-2013 | 2013-2014 | 2014-2015 | 2015-2016 | 2016-2017 | 2017-2018 | 2018-2019 |  |
| Low-calorie sweetened food and beverage consumption (g/day) | | | | | | | | | | | |  |
| Low-LCS | 51.5  (0.5,150.0) | 62.0  (0.5,225.0) | 72.5  (0.5,239.3) | 51.6  (0.6,176.7) | 51.8  (0.3,166,7) | 52.2  (0.8,210.8) | 57.0  (0.0,175.0) | 50.0  (0.3,260.0) | 50.0  (0.5,175.0) | 62.5  (0.8,200.0) | 62.5  (0.5,340.8) | 0.5 |
| Mid-LCS | 187.75  (67.3,568.0) | 194.0  (74.3,524.4) | 197.4  (71.0,522.8) | 187.5  (87.5,434.5) | 200.0  (62.5,406.5) | 196.0  (50.0,551.0) | 175  (66.7,526.7) | 196.7  (78.6,703.1) | 175.0  (75.0,400.0) | 201.4  (75.0,473.8) | 200.0  (71.0,495.0) | 1.0 |
| High-LCS | 448.9  (187.5,1568.2) | 453.0  (170.0,2645.9) | 456.6  (197.0,1628.0) | 425.0 (100.0,1463.4) | 537.5  (162.5,2585.0) | 457.4  (165.0,1328.0) | 482.3  (156.3,1687.5) | 476.4  (150.0,1898.7) | 412.5  (150.0,1750.0) | 508.8  (142.0,4395.8) | 470.6  (162.5,2450.0) | 0.3 |
| Low-calorie sweetened food and beverage as percentages of total food and beverage intake (%g_total_/d) | | | | | | | | | | | |  |
| Low-LCS | 3.3  (0.0,6.7) | 3.4  (0.0,6.8) | 3.8  (0.0,6.6) | 3.6  (0.1,6.8) | 3.5  (0.0,6.7) | 3.5  (0.0,6.7) | 3.5  (0.0,6.7) | 3.7  (0.0,6.7) | 3.8  (0.0,6.6) | 3.7  (0.0,6.8) | 3.6  (0.0,6.7) | 0.3 |
| Mid-LCS | 11.2  (6.8,17.4) | 12.0  (6.8,17.4) | 11.7  (6.7,17.4) | 11.9  (6.8,17.4) | 12.4  (6.8,17.4) | 11.4  (6.8,17.4) | 11.2  (6.8,17.4) | 12.0  (6.9,17.2) | 10.5  (6.9,17.3) | 11.9  (6.8,17.3) | 11.5  (6.9,17.3) | 0.6 |
| High-LCS | 25.9  (17.4,58.4) | 26.0 (17.5,72.9) | 28.6  (17.4,64.7) | 26.4  (17.4,61.0) | 28.8  (17.5,71.1) | 26.5  (17.4,66.1) | 29.4  (17.4,71.0) | 29.1  (17.5,64.7) | 26.2  (17.4,64.4) | 27.5  (17.6,83.3) | 28.5  (17.5, 73.0) | 0.2 |
| Low-calorie sweetened beverages (g/day) | | | | | | | | | | | |  |
| Low-LCS | 50.0  (0.0,150.0) | 50.0  (0.0,166.7) | 70.9  (0.0,239.25) | 50.0  (0.0,176.7) | 50.0  (0.0,166.7) | 50.0  (0.0,210.8) | 50.0  (0.0,175.0) | 50.0  (0.0,260.0) | 50.0  (0.0,175.0) | 62.5  (0.0,200.0) | 62.5  (0.0,335.8) | 1.0 |
| Mid-LCS | 173.5  (62.5,568.0) | 188.6  (0.0,497.0) | 182.5  (71.0,522.8) | 180.3  (0.0,434.5) | 200.0  (62.5,396.5) | 187.5  (50.0,551.0) | 175.0  (62.5,522.0) | 192.3  (78.6,703.1) | 175.0  (67.5,400.0) | 200.0  (0.0,457.5) | 200.0  (50.0,495.0) | 0.8 |
| High-LCS | 440.8  (187.5,1562.7) | 447.0  (150.0,2645.0) | 450.0  (167.0,1628.0) | 421.5  (100.0,1463.4) | 537.5  (162.5,2585.0) | 453.1  (165.0,1328.0) | 474.0  (156.2,1687.5) | 475.1  (150.0,1898.7) | 412.5  (150.0,1750.0) | 500  (142.0,4395.8) | 426.5  (162.5,2450.0) | 0.2 |
| Low-calorie sweetened beverage as percentages of total beverage intake (%g_total_/d) | | | | | | | | | | | |  |
| Low-LCS | 5.0  (0.0,13.8) | 5.6  (0.0,17.6) | 6.9  (0.0,21.2) | 6.1  (0.0,16.3) | 6.00  (0.0,14.7) | 5.7  (0.0,20.0) | 6.1  (0.0,22.6) | 5.9  (0.0,17.7) | 6.4  (0.0,14.4) | 6.3  (0.0,14.8) | 5.8  (0.0,17.3) | 0.3 |
| Mid-LCS | 20.5  (8.7,49.9) | 21.3  (0.0,45.9) | 21.8  (7.4,54.5) | 20.0  (0.0,38.0) | 21.3  (10.6,43.4) | 19.3  (4.5,47.7) | 18.7  (8.0,48.9) | 20.5  (4.8,61.1) | 19.7  (9.4,39.5) | 21.7  (0.0,49.1) | 19.5  (9.0,36.2) | 0.2 |
| High-LCS | 45.4  (25.1,90.2) | 46.5  (24.6,100.0) | 49.9  (28.8,98.0) | 46.7  (23.0,93.3) | 52.4  (23.9,95.7) | 47.2  (21.2,99.4) | 52.0  (18.5,100.0) | 48.3  (25.3,99.3) | 43.6  (21.3,96.6) | 50.4  (18.4,99.6) | 49.2  (25.4,100.0) | 0.3 |

**Statistical analysis:** Levels of LCS product consumption across years were compared using quantile regression.

**Appendix Table 3 Association between daily LCS product consumption and dietary components from 2008-2019 (n = 5,922)**

| Intake of nutrients | Free sugar intake  (g/d) | Free sugar intake from beverages  (g/d) | Total sugar intake  (g/d) | Total sugar intake  (%kcal_total_/d) | Total sugar intake from beverages  (g/d) | Total sugar intake from beverages  (%kcal_drinks_/d) |
| --- | --- | --- | --- | --- | --- | --- |
|  | Coefficient (95% CI) | Coefficient (95% CI) | Coefficient (95% CI) | Coefficient (95% CI) | Coefficient (95% CI) | Coefficient (95% CI) |
| LCS product consumption at year 1**^+^**   - No-LCS - Low-LCS - Mid-LCS - High-LCS | Reference  7.8 (2.6, 13.0)*  0.8 (-4.4, 6.0)  -7.2 (-11.8, -2.7)* | Reference  1.7 (-2.1, 5.6)  -3.9 (-7.7, -0.2)*  -11.6 (-14.9, -8.4)*^+^ | Reference  10.7 (4.6, 16.9)*  1.4 (-4.6, 7.4)  -9.5 (-14.7, -4.2)* | Reference  0.5 (-0.6, 1.6)  -0.7 (-1.7, 0.3)  -2.4 (-3.4, -1.5)*^+^ | Reference  2.2 (-1.8, 6.3)  -3.6 (-7.6, 0.4)  -13.4 (-16.8, -10.0)*^+^ | Reference  -0.3 (-1.2, 0.6)  -1.4 (-2.2, -0.5)*  -3.4 (-4.2, -2.6)*^+^ |
| Survey year | -2.2 (-2.7, -1.7)*^+^ | -1.9 (-2.3, -1.5)*^+^ | -2.1 (-2.7, -1.6)*^+^ | -0.4 (-0.5, -0.3)*^+^ | -2.0 (-2.4, -1.6)*^+^ | -0.5 (-0.6, -0.4)*^+^ |
| Interaction   - No-LCS x Year - Low-LCS x Year - Mid-LCS x Year - High-LCS x Year | Reference  -0.6 (-1.5, 0.2)  -1.3 (-0.7, 1.0)  -0.4 (-0.3, 1.1) | Reference  -0.2 (-0.8, 0.4)  0.4 (-0.2, 1.0)  0.8 (0.3, 1.3)* | Reference  -0.9 (-1.8, 0.1)  -0.1 (-1.1, 0.8)  0.1 (-0.8, 0.9) | Reference  -0.02 (-0.2, 0.2)  0.1 (-0.1, 0.3)  0.2 (0.01, 0.3)*^+^ | Reference  -0.1 (-0.8, 0.5)  0.4 (-0.3, 1.0)  0.9 (-0.3, 1.4)*^+^ | Reference  0.1 (-0.1, 0.2)  0.2 (0.03, 0.3)*  0.3 (0.1, 0.4)*^+^ |

| Intake of dietary components | Water intake  (g/d) | Ultra-processed food and beverage intake (g/d) | Ultra-processed intake  (g/d) | Minimally processed food and beverage intake (g/d) |
| --- | --- | --- | --- | --- |
|  | Coefficient (95% CI) | Coefficient (95% CI) | Coefficient (95% CI) | Coefficient (95% CI) |
| LCS product consumption at year 1**^+^**   - No-LCS - Low-LCS - Mid-LCS - High-LCS | Reference  -17.1 (-89.1, 54.9)  -71.6 (-128.3, -15.0)*  -185.5 (-237.6, -133.5)*^+^ | Reference  118.9 (72.4, 165.3)*^+^  216.2 (162.8, 269.5)*^+^  474.9 (422.8, 527.0)*^+^ | Reference  64.8 (43.1, 86.5)*^+^  47.0 (23.9, 70.1)*  53.0 (33.1, 72.9)*^+^ | Reference  -1.2 (-88.0, 85.6)  -130 (-205.2, -54.9)*  -334 (-405.8, -262.5)*^+^ |
| Survey year | 28.9 (21.1, 36.8)*^+^ | -11.1 (-16.3, -6.0)*^+^ | -1.9 (-4.2, 0.3) | 21.0 (11.1, 30.9)*^+^ |
| Interaction   - No-LCS x Year - Low-LCS x Year - Mid-LCS x Year - High-LCS x Year | Reference  8.6 (-8.4, 25.7)  -9.4 (-20.1, 1.3)  -17.4 (-26.5, -8.,4)*^+^ | Reference  -5.3 (-13.1, 2.6)  0.2 (-8.4, 8.9)  5.6 (-3.7, 14.8) | Reference  -5.9 (-9.5, -2.4)*  -1.3 (-5.1, 2.4)  -2.7 (-6.1, 0.7) | Reference  7.5 (-10.0, 25.1)  -8.8 (-22.1, 4.4)  -17.3 (-29.6, -5.0)* |

* P-value < 0.05 before Bonferroni correction, *^+^ P-value < 0.05 after Bonferroni correction

**^+^** The levels of LCS product consumption represent nutritional outcome in children relative to group 1in 2008-2009.

**Statistical analysis:** Multivariable linear regression adjusted for age, sex, ethnicity, household income, Indices of Multiple Deprivation, and body mass index

**Appendix Table 4 Energy and sugar intake by LCS product consumption levels among UK children (2008/09 and 2018/19)**

| Intake of energy and sugar | Free sugar intake  (%kcal_total_/d) | | Free sugar intake  (g/d) | | Free sugar intake from beverages  (%kcal_drinks_/d) | | Free sugar intake from beverages (g/d) | | Total energy intake  (kcal/d) | |
| --- | --- | --- | --- | --- | --- | --- | --- | --- | --- | --- |
|  | Year 1  Coefficient (95% CI) | Year 11  Coefficient  (95% CI) | Year 1  Coefficient (95% CI) | Year 11  Coefficient (95% CI) | Year 1  Coefficient (95% CI) | Year 11  Coefficient (95% CI) | Year 1  Coefficient (95% CI) | Year 11  Coefficient (95% CI) | Year 1  Coefficient (95% CI) | Year 11  Coefficient (95% CI) |
| - No-LCS  - Low-LCS  - Mid-LCS  - High-LCS | Reference  0.3  (-0.7, 1.3)  -0.7  (-1.7, 0.3)  -1.9  (-2.8, -1.0) *^+^ | Reference  0.3  (-0.7, 1.3)  0.8  (-0.2, 1.8)  0.03  (-0.8, 0.9) | Reference  7.8  (2.6, 13.0)*  0.8  (-4.4, 6.0)  -7.2  (-11.8,-2.7)* | Reference  1.3  (-3.6, 6.3)  2.1  (-2.8, 7.0)  -3.6  (-7.8, 0.6) | Reference  -0.3  (-1.2, 0.6)  -1.4  (-2.2, -0.6)*  -3.0  (-3.7, -2.2) *^+^ | Reference  -0.1  (-1.0, 0.7)  0.2  (-0.6, 1.0)  -0.7  (-1.4, -0.04) | Reference  1.7  (-2.1, 5.6)  -3.9  (-7.7, -0.2)*  -11.6  (-14.9, -8.4) *^+^ | Reference  -0.5  (-4.1, 3.1)  -0.1  (-3.3, 3.1)  -3.8  (-6.6, -1.1)* | Reference  162.4  (98.3,226.5) *^+^  73.8  (2.9, 144.7) *^+^  18.6  (-42.3, 79.4) | Reference  14.3  (-49.6, 78.2)  -8.4  (-80.4, 63.6)  -104.5  (-167.8,-41.2) *^+^ |

| Intake of sugar | Total sugar intake  (%kcal_total_/d) | | Total sugar intake  (g/d) | | Total sugar intake from beverages  (%kcal_drinks_/d) | | Total sugar intake from beverages  (g/day) | |
| --- | --- | --- | --- | --- | --- | --- | --- | --- |
|  | Year 1  Coefficient (95% CI) | Year 11  Coefficient (95% CI) | Year 1  Coefficient (95% CI) | Year 11  Coefficient (95% CI) | Year 1  Coefficient (95% CI) | Year 11  Coefficient (95% CI) | Year 1  Coefficient (95% CI) | Year 11  Coefficient (95% CI) |
| - No-LCS  - Low-LCS  - Mid-LCS  - High-LCS | Reference  0.5  (-0.6, 1.6)  -0.7  (-1.7, 0.3)  2.4  (-3.4, -1.5) *^+^ | Reference  0.3 (-0.7, 1.3)  0.2  (-0.8, 1.2)  -0.9 (-1.8, 0.1) | Reference  10.7  (4.6, 16.9)*  1.4  (-4.6, 7.4)  -9.5  (-14.7, -4.2)* | Reference  2.2  (-3.4, 7.8)  -0.1  (-5.6, 5.4)  -8.7  (-13.6, -3.7)* | Reference  -0.3  (-1.2, 0.6)  -1.4  (-2.2, -0.5)*  -3.4  (-4.2, -2.6) *^+^ | Reference  0.2  (-0.6, 1.1)  0.3  (-0.5, 1.1)  -0.7  (-1.5, -0.03) | Reference  2.2  (-1.8, 6.3)  -3.6  (-7.6, 0.4)  -13.4  (-16.8,-10.0) *^+^ | Reference  1.0  (-2.8, 4.8)  -0.04  (-3.5, 3.4)  -4.9  (-7.9, -1.8)* |

* P-value < 0.05 before Bonferroni correction, *^+^ P-value < 0.05 after Bonferroni correction

**^+^** The levels of LCS product consumption represent nutritional outcome in children relative to group1 in 2008-2009.

**Statistical analysis:** Multivariable linear regression adjusted for age, sex, ethnicity, household income, Indices of Multiple Deprivation, and body mass index

**Appendix Table 5 Other dietary component intake by LCS product consumption levels among UK children (2008/09 and 2018/19)**

| Intake of water and ultra-processed food and beverages | Water intake (%g_drinks_/d) | | Water intake (g/d) | | Ultra-processed food and beverage intake (%g_total_/d) | | Ultra-processed food and beverage intake (g/d) | |
| --- | --- | --- | --- | --- | --- | --- | --- | --- |
|  | Year 1  Coefficient  (95% CI) | Year 11  Coefficient  (95% CI) | Year 1  Coefficient  (95% CI) | Year 11  Coefficient  (95% CI) | Year 1  Coefficient  (95% CI) | Year 11  Coefficient  (95% CI) | Year 1  Coefficient  (95% CI) | Year 11  Coefficient  (95% CI) |
| - No-LCS  - Low-LCS  - Mid-LCS  - High-LCS | Reference  -0.4  (-4.3, 3.5)  -7.3  (-11.0, -3.8) *^+^  -19.6  (-22.7, -16.6) *^+^ | Reference  -4.5  (-8.8, -0.3)*  -19.7  (-24.0, -15.5) *^+^  -37.0  (-40.6, -33.4) *^+^ | Reference  -17.1  (-89.1, 54.9)  -71.6  (-128.3, -15.0)*  -185.5  (-237.6, -133.5)*^+^ | Reference  69.2  (-47.4, 185.8)  -165.6  (-234.3, -96.9)*^+^  -359.9  (-415.4, -304.4)*^+^ | Reference  3.4  (0.8, 5.8)*  9.6  (7.3, 12.0)*^+^  22.4  (20.1, 24.6) *^+^ | Reference  1.8 (-0.6, 4.2)  12.7  (10.3, 15.1)*^+^ 29.1  (26.6, 31.6)*^+^ | Reference  118.9  (72.4, 165.3)*^+^  216.2  (162.8, 269.5)*^+^  474.9  (422.8, 527.0)*^+^ | Reference  66.3  (17.8, 114.8)*^+^  218.6  (168.9, 268.2)*^+^  530.7  (471.4, 590.0)*^+^ |

| Intake of ultra-processed food and beverages and minimally processed foods and beverages | Ultra-processed food intake  (%g_total_/d) | | Ultra-processed food intake  (g/d) | | Minimally processed food and beverage intake (%g_total_/d) | | Minimally processed food and beverage intake (g/d) | |
| --- | --- | --- | --- | --- | --- | --- | --- | --- |
|  | Year 1  Coefficient  (95% CI) | Year 11  Coefficient  (95% CI) | Year 1  Coefficient  (95% CI) | Year 11  Coefficient  (95% CI) | Year 1  Coefficient  (95% CI) | Year 11  Coefficient  (95% CI) | Year 1  Coefficient  (95% CI) | Year 11  Coefficient  (95% CI) |
| - No-LCS  - Low-LCS  - Mid-LCS  - High-LCS | Reference  1.5  (0.2, 2.8)*  1.4  (0.1, 2.8)*  1.0  (-0.3, 2.2) | Reference  -0.8  (-2.2, 0.6)  1.9  (0.5, 3.3)*  1.2  (-0.2, 2.6) | Reference  64.8  (43.1, 86.5)*^+^  47.0  (23.9, 70.1)*  53.0  (33.1, 72.9)*^+^ | Reference  5.4  (-15.5, 26.2)  33.6  (11.6, 55.7)*  26.1  (5.1, 47.0)* | Reference  -2.9  (-5.3, -0.4)*  -9.1  (-11.4, -6.7)*^+^  -21.7  (-23.9, -19.4)*^+^ | Reference  -1.9  (-4.3, 0.5)  -12.6  (-15.0, -10.2)*^+^  -28.5  (-31.0, -26.0)*^+^ | Reference  -1.2  (-88.0, 85.6)  -130  (-205.2, -54.9)*  -334  (-405.8, -262.5)*^+^ | Reference  74.2  (-41.3, 189.6)  -218.2  (-301.7, -134.8)*^+^  -507.2  (-581.9, -432.4)*^+^ |

* P-value < 0.05 before Bonferroni correction, *^+^ P-value < 0.05 after Bonferroni correction

**^+^** The levels of LCS product consumption represent nutritional outcome in children relative to group1 in 2008-2009.

**Statistical analysis:** Multivariable linear regression adjusted for age, sex, ethnicity, household income, Indices of Multiple Deprivation, and body mass index

Total foods and beverages

= 5196 items

Recorded with a generic name = 4324 items

Recorded with a trade name

= 872 items

18 items identified as LCS products

(17 foods and one beverage)

Searched through dessert, confectionery, and sweetened dairy products categories for descriptors^2^ that contain “sugar free” or “diabetic”

Searched supermarket catalogues for the ingredient list^3^ of similar items with a descriptor such as "low sugar," "low calorie," or "sweetener"^4^

Searched the generic name for items recorded with “artificial sweetener” or similar descriptors^1^

Screened the remaining items^5^

Non-LCS products

= 4264 items

Searched the ingredient lists for use of LCS

Non-LCS products

= 792 items

8 items identified as LCS products

(8 foods)

33 items identified as LCS products

(12 foods and

21 beverages)

80 items identified as LCS products

(15 foods and

65 beverages)

1 item identified as LCS product

(Whey protein)

**Appendix Fig. 1 Flow chart for the identification of LCS products in the National Diet and Nutrition Survey**

^1^e.g., yogurt with fruit and artificial sweetener”, and “meatballs in tomato sauce with artificial sweetener”

^2^e.g., custard, gum, candy, chocolate, and yogurt

^3^The ingredients of these products were searched. They were classified as LCS products if at least one brand of those products in the market contains LCS.

^4^e.g., soup, sauce, carbonated drinks, and fruit drinks

^5^All products identified with detailed descriptor in generic names, such as “mineral water with herbal extract”, and “fruit juice drink no added sugar”, underwent a search for their ingredient lists. They were classified as LCS products if at least one brand of those products in the market contains LCS.

**
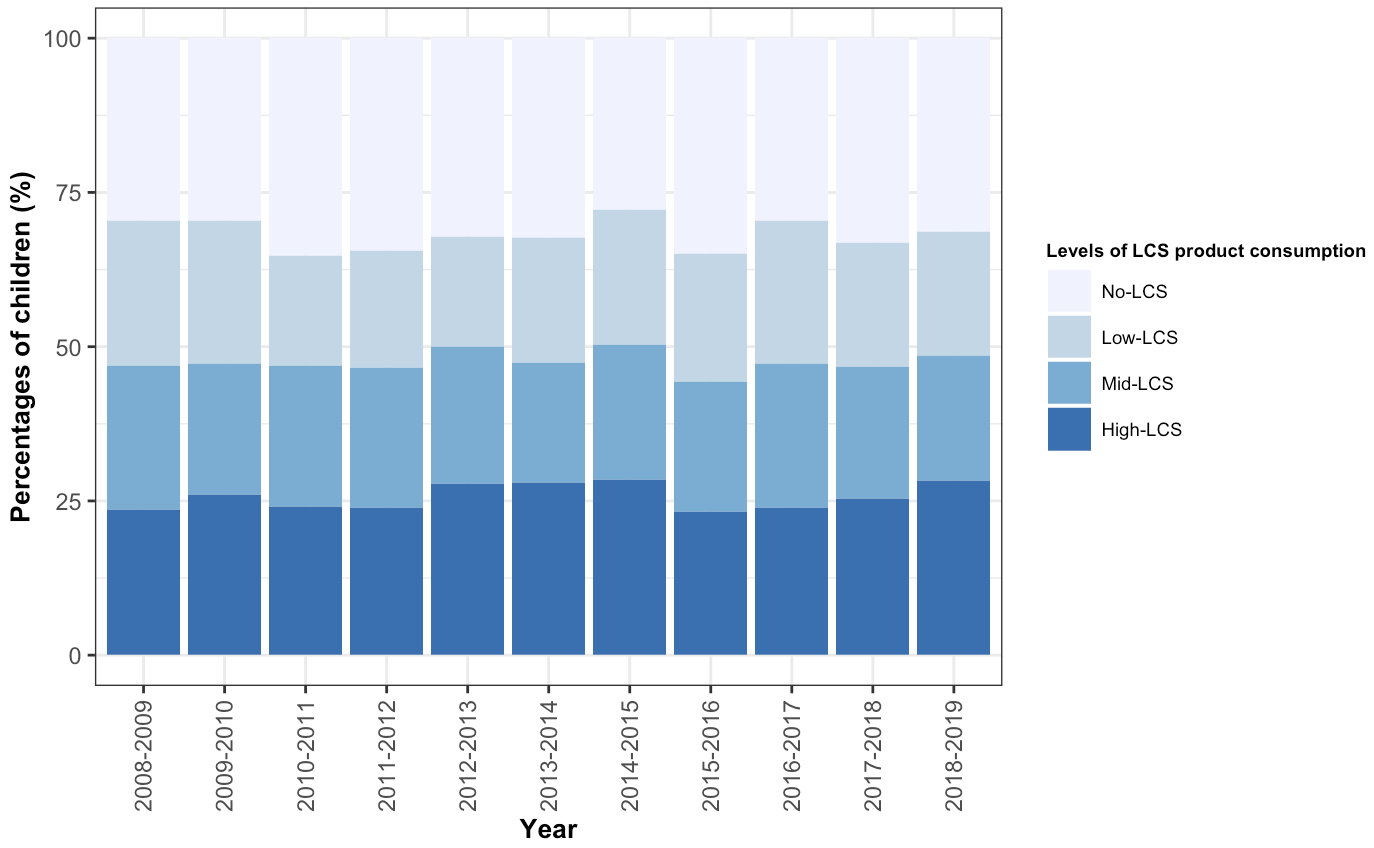
**

**Appendix Fig. 2 Trends in proportion of UK children across levels of LCS product consumption from 2008-2019**

**
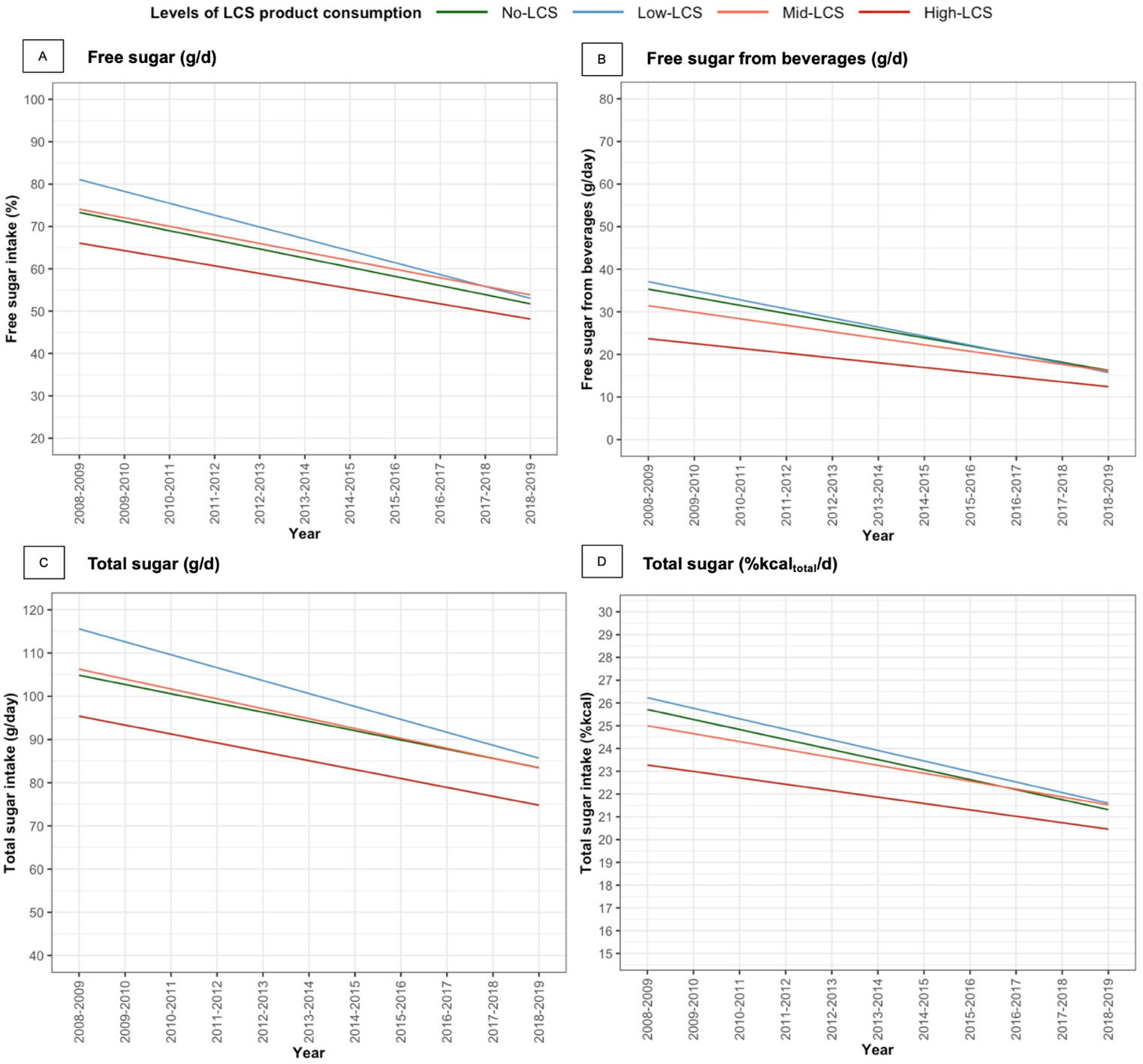
**

**Appendix Fig. 3 Trends in daily dietary component intake by levels of LCS product consumption among UK children**


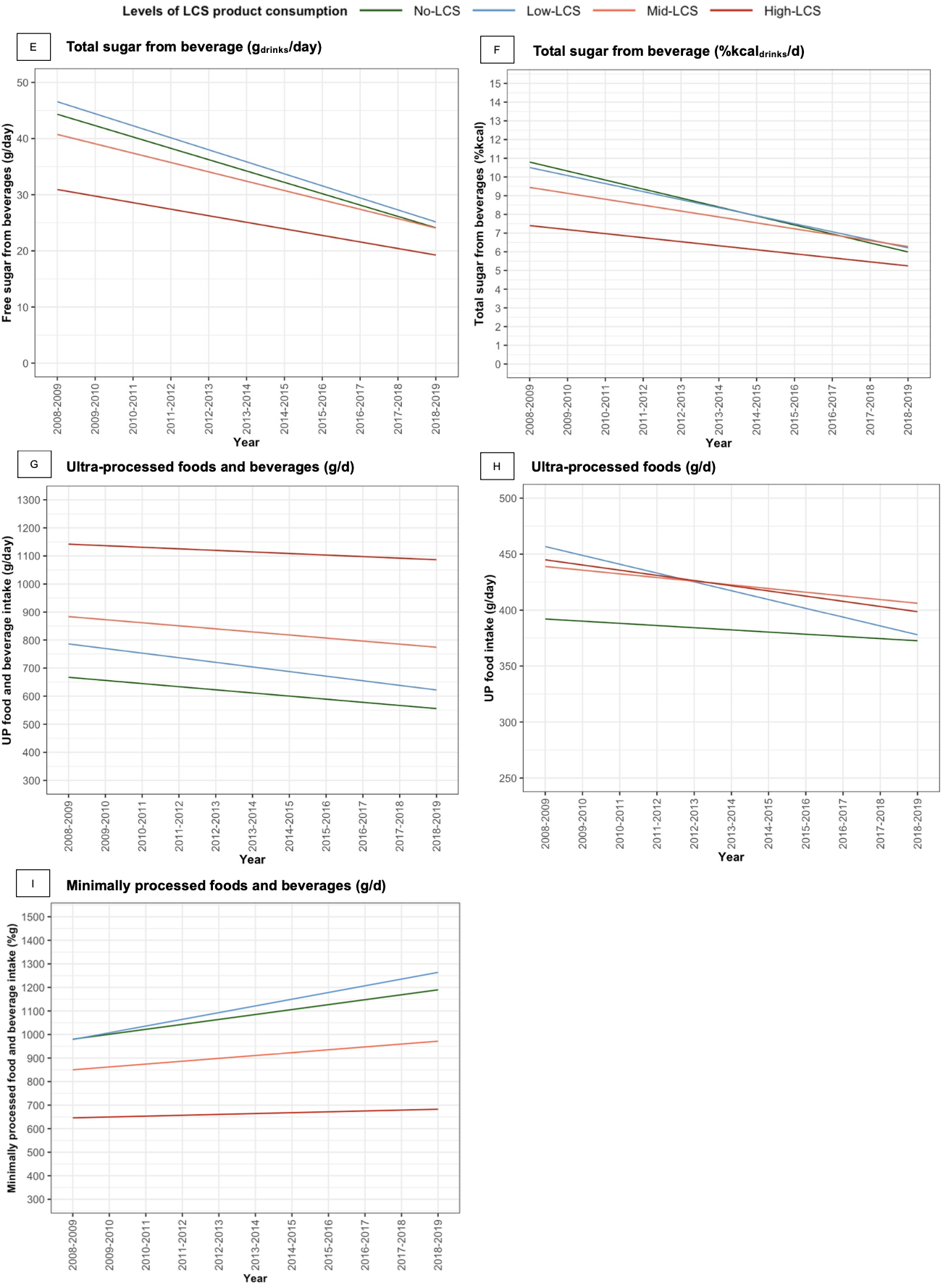
**Appendix Fig. 3 (cont.) Trends in daily dietary component intake by levels of LCS product consumption among UK children**
